# Exploring the inhibitory effect and mechanism of 3,4,5-trihydroxybiphenyl on α-glucosidase: an integrated experimental and computational approach

**DOI:** 10.3389/fphar.2025.1584264

**Published:** 2025-07-03

**Authors:** Ruofan Guo, Guohua Yu, Yuan Li, Youyou Wang, Huixia Fan, Shuo Zhang, Chen Wang, Junhui Zhou, Jian Yang, Feng Gao, Zhiqiang Luo

**Affiliations:** ^1^ State Key Laboratory for Quality Ensurance and Sustainable Use of Dao-di Herbs, National Resource Center for Chinese Materia Medica, China Academy of Chinese Medical Sciences, Beijing, China; ^2^ Xiyuan Hospital of China Academy of Chinese Medical Sciences, Beijing, China; ^3^ School of Life Sciences, Beijing University of Chinese Medicine, Beijing, China; ^4^ Evaluation and Research Center of Daodi Herbs of Jiangxi Province, Ganjiang, China; ^5^ School of Traditional Chinese Medicine, Beijing University of Chinese Medicine, Beijing, China; ^6^ School of Chinese Materia Medica, Beijing University of Chinese Medicine, Beijing, China; ^7^ Hebei Baicaokangshen Pharmaceutical Co., Ltd, Shenzhou, China

**Keywords:** α-glucosidase, inhibition activity, interaction mechanism, postprandial blood glucose, diabetes

## Abstract

3,4,5-Trihydroxybiphenyl (THB) is a naturally occurring compound derived from *Sorbus pohuashanensis*, primarily reported for its antifungal activity. However, its potential to inhibit α-glucosidase remains unclear. In this study, we assessed the inhibitory effects of THB on α-glucosidase and explored the mechanism of inhibition through kinetic analysis, multispectral techniques, molecular docking and molecular dynamics simulations. Furthermore, a sucrose tolerance test was performed to evaluate the effects of THB on postprandial blood glucose (PBG) levels in mice. The results showed that THB exhibited a non-competitive and reversible inhibitory effect on α-glucosidase, with IC_50_, K_m_, and K_i_ values of 11.52 μM, 0.69 ± 0.02 mM, and 26.26 ± 4.95 μM, respectively. THB showed a good affinity for α-glucosidase, with a K_D_ value of 3.91 × 10^−5^ M. The interaction between THB and α-glucosidase induced significant changes in the enzyme’s microenvironment and secondary structure. The primary driving force for the binding of THB to α-glucosidase was hydrogen bonding. Additionally, THB could significantly reduce PBG levels in mice. Collectively, these findings suggest that THB holds potential as a natural inhibitor for the development of α-glucosidase-targeting agents.

## 1 Introduction

Type 2 diabetes mellitus (T2DM) is an escalating global health issue with a rapidly increasing prevalence (ElSayed et al., 2023). Current epidemiological projections estimate that the number of individuals affected by diabetes could reach a staggering 700 million worldwide by 2045 (Du et al., 2024). The defining clinical characteristic of T2DM is chronic hyperglycemia—elevated blood glucose levels—which can lead to a wide range of debilitating complications (Xu et al., 2024). Consequently, effective management of hyperglycemia is a cornerstone of T2DM treatment. Among the pharmacological options available, α-glucosidase inhibitors represent a particularly noteworthy class of antidiabetic drugs (Daou et al., 2022). These agents work by reversibly inhibiting α-glucosidase located in the gut, thereby reducing the hydrolysis of carbohydrates into glucose and mitigating postprandial hyperglycemia (Liu et al., 2021). Despite their clinical use since 1990, only acarbose, miglitol and voglibose are currently marketed (Mushtaq et al., 2023). However, their use is often limited by gastrointestinal adverse reactions, primarily flatulence and diarrhea, which frequently lead to treatment discontinuation among patients (Jiang et al., 2024). Recently, increasing attention has been directed toward natural bioactive compounds that exhibit similar hypoglycemic effects to acarbose but with minimal or no adverse reactions (Dirir et al., 2021). These compounds hold significant potential as safe and effective alternatives for the prevention and treatment of diabetes.

**FIGURE 1:**
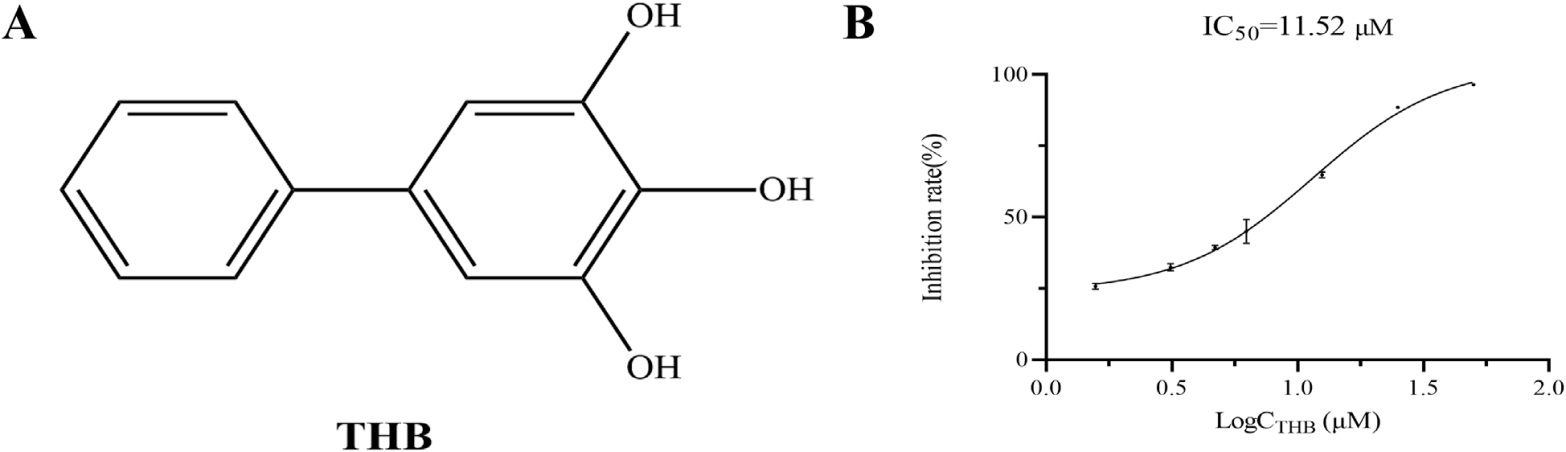
**(A)** Chemical structure of THB; **(B)** α-Glucosidase inhibition by THB (*n* = 3).

Biphenyl derivatives are extensively distributed across various plants and exhibit a broad spectrum of biological activities, including antifungal, antibacterial, anticancer, antidiabetic, and anti-inflammatory effects (Singh et al., 2023). In recent years, natural biphenyls such as magnolol and honokiol have demonstrated potent α-glucosidase inhibitory activity (Pulvirenti et al., 2017). 3,4,5-Trihydroxybiphenyl (THB, Figure 1A), a naturally occurring biphenyl compound derived from *Sorbus pohuashanensis*, has primarily been studied for its antifungal properties (Song et al., 2021). However, to the best of our knowledge, the potential inhibitory effects of THB on α-glucosidase have yet to be explored.

The aim of this study was to explore the inhibitory effects of 3,4,5-Trihydroxybiphenyl (THB) on α-glucosidase and to elucidate the mechanism of inhibition using kinetic analysis, multispectral techniques (including circular dichroism and surface plasmon resonance), molecular docking and molecular dynamics simulations. Additionally, a sucrose tolerance experiment was conducted to validate its effects on postprandial blood glucose (PBG) levels in mice. Overall, the findings from this research would provide valuable insights into the potential development of THB as a therapeutic agent for the prevention and treatment of type 2 diabetes mellitus.

## 2 Materials and methods

### 2.1 Materials

α-Glucosidase and acarbose were obtained from MedChemExpress (Monmouth Junction, NJ, United States). P-Nitrophenyl-α-D-glucoside (p-NPG) was sourced from Shanghai Yuanye Bio-Technology Co., Ltd (Shanghai, China). THB was isolated from *S. pohuashanensis* leaves by our research group, and its structural identification has been detailed in our previous studies (Song et al., 2021).

### 2.2 *In vitro* α-glucosidase inhibition assay

The α-glucosidase inhibitory activity was assessed using a previously described method (Wang et al., 2019). THB solutions (2 μL) at various concentrations were prepared in a phosphate buffer (PBS, pH 6.8) and mixed with α-glucosidase (20 μL) in a 96-well microplate. The reaction was allowed to proceed for 15 min. Subsequently, 20 μL of a 10 mmol/L pNPG substrate solution was added, and the mixture was incubated for another 30 min. The reaction was then terminated by adding 80 μL of 0.2 mol/L Na_2_CO_3_. Absorbance was recorded at 405 nm using a microplate reader ((Molecular Devices, LLC., CA, United States).

### 2.3 The inhibition kinetics of THB on α-glucosidase

The protocol for the inhibition kinetics test mirrored that of the α-glucosidase inhibition assay. In terms of enzyme kinetics, absorbance was measured for mixtures with varying concentrations of THB and α-glucosidase. These measurements were then plotted against the concentrations of α-glucosidase to evaluate THB’s reversibility. For substrate kinetics, absorbance was recorded for mixtures with different THB and substrate concentrations (Kaur et al., 2021). Lineweaver-Burk plots were utilized to determine the inhibition type and to calculate the inhibition constant (Ki). The Ki for noncompetitive inhibition was calculated using the Equations 1-4:

$$\frac{1}{v}=\frac{K_{m}}{V_{\max}}\left(1+\frac{\left[I\right]}{K_{i}}\right)\frac{1}{\left[S\right]}+\frac{1}{V_{\max}}\left(1+\frac{\left[I\right]}{\alpha K_{i}}\right)$$ (1)

$$\text{Slope}=\frac{K_{m}}{V_{\max}}\left(1+\frac{\left[I\right]}{K_{i}}\right)$$ (2)

$$Y‐\text{intercept}=\frac{1}{V_{\max}}\left(1+\frac{\left[I\right]}{\alpha K_{i}}\right)=\frac{1}{V_{\max}^{\text{app}}}$$ (3)

$$V^{\prime }\max=\frac{\text{Vmax}}{1+\frac{\left[I\right]}{\text{Ki}}}$$ (4)

In these equations, v represents initial reaction velocity [I] denotes inhibitor concentration, and [S] indicates substrate concentration. The symbol α signifies the ratio of noncompetitive to competitive inhibition constants, equaling one for noncompetitive inhibition.

### 2.4 The inactivation kinetics of THB on α-glucosidase

The real-time inactivation kinetics of THB on α-glucosidase were monitored at various intervals. In brief, mixtures containing α-glucosidase (10 μg ⋅ mL^-1^), PBS buffer, and THB solutions at different concentrations (1.57–9.38 μM) was prepared in 96-well plates. These mixtures were then incubated at 37°C for durations of 0, 15, 30, 60, 120, and 180 min, respectively. After pre-incubation, 20 μL of 10 mM P-NPG solution was introduced, and the reaction proceeded for 30 min. Absorbance at 405 nm was immediately measured. The steady-state rate of substrate hydrolysis, represented as (V_t_)/(V_0_), was plotted on the vertical axis against the corresponding pre-incubation time on the horizontal axis.

### 2.5 Synergistic inhibition of α-glucosidase by THB and acarbose

The interaction between THB and acarbose in inhibiting α-glucosidase was evaluated using the combination index (CI) approach. CI values were calculated using CompuSyn software (CompuSyn, Inc., NJ, United States). CI values are calculated using formula 5:

$$\text{CI}=\frac{{\left(D\right)}_{1}}{{\left(D_{X}\right)}_{1}}+\frac{{\left(D\right)}_{2}}{{\left(D_{X}\right)}_{2}}$$ (5)

In this equation (D_x_)_1_ and (D_x_)_2_ represent the concentrations of THB and acarbose, respectively, that produce a specific inhibitory effect when used individually (D)_1_ and (D)_2_ denote the concentrations of THB and acarbose, respectively, that achieve the same inhibitory effect when used in combination. The nature of the interaction between acarbose and THB was determined based on the CI value. A CI exceeding 1.1 indicates antagonism, while a value between 0.9 and 1.1 suggests an additive effect. Synergistic inhibition is characterized by a CI below 0.9.

### 2.6 Surface plasmon resonance (SPR) analysis

A Biacore 8 K instrument (GE Healthcare, Sweden) was employed for SPR analysis. The CM5 sensor chip surface underwent activation with an NHS/EDC mixture, after which α-glucosidase was immobilized using an Amine Coupling Kit. Affinity measurements were performed in accordance with the manufacturer’s guidelines. To account for non-specific binding, a reference channel lacking conjugated protein was incorporated (Luo et al., 2023; Luo et al., 2025). Analytes were introduced at various concentrations (6.25–100 μM) with a constant flow rate of 30 μL/min. The association-dissociation process was monitored in real-time by observing changes in response values. Biacore Insight Evaluation Software was utilized to fit the SPR curves, employing a 1:1 Langmuir binding model for the determination of binding constants and kinetic parameters.

### 2.7 Circular dichroism

Circular dichroism (CD) spectra were obtained using a Jasco J-810 spectrophotometer. To examine alterations in secondary structure, measurements were taken in the far-UV range (190–260 nm). The instrument was set with a path length of 1 nm and operated at a scanning rate of 50 nm/min. For the analysis, THB concentration was fixed at 3 mM, while α-glucosidase was maintained at 0.05 μg/μL. CDNN software was employed to quantify the modifications in the secondary structure components of α-glucosidase based on the CD data.

### 2.8 Molecular docking

To elucidate the binding mechanism between THB and α-glucosidase, molecular docking simulations were performed using AutoDock 4.2 (https://autodock.scripps.edu/). The crystal structure of *Saccharomyces cerevisiae* α-glucosidase (PDB ID: 3A4A, 1.60 Å resolution, wild-type) was retrieved from the Protein Data Bank (https://www.rcsb.org/) (Yamamoto et al., 2010). Prior to docking, the protein structure was prepared by removing water molecules, adding hydrogen atoms, assigning atomic charges, and eliminating co-crystallized ligand (maltose). The three-dimensional structure of THB was constructed using ChemDraw 3D and energy-minimized with Open Babel. In the docking simulations, α-glucosidase was treated as a rigid receptor, while the THB ligand was allowed full flexibility. The active site was predicted using the Site Finder tool in MOE (Molecular Operating Environment) software (Riyaphan et al., 2021). Potential binding conformations were explored using the Lamarckian genetic algorithm (LGA) implemented in AutoDock. The most favorable binding pose was selected based on the lowest docking score observed between the enzyme and the ligand. Further analysis with BIOVIA Discovery Studio 2021 characterized key interaction features, including hydrogen bonds, hydrophobic contacts, and other critical binding site interactions.

### 2.9 Molecular dynamics (MD) simulation

The most favorable docking pose of THB-α-glucosidase complexes, identified through molecular docking, was selected as the starting conformation for MD analysis. All simulations were performed using GROMACS V2020 with the AMBER03 force field, where ligand topologies were generated by Sobtop software (http://sobereva.com/soft/Sobtop). A simulation box was constructed, centering the protein-ligand complex and ensuring a minimum distance of 10 Å from the box edge. The system was filled with TIP3P water molecules and neutralized by adding 16 Na^+^ ions. Energy minimization was performed to relax the system into a local energy minimum, with a maximum of 50,000 iterations and a convergence threshold set to 10 kJ/mol/nm as the default. Subsequently, the system underwent a two-step equilibration process: first, a 1 ns canonical ensemble (NVT) simulation, followed by a 1 ns isothermal-isobaric (NPT) simulation. Both steps were conducted at 310.15 K and 1.0 ATM. Finally, we carried out a 200 ns molecular dynamics simulation using the equilibrated system, and the resulting trajectories were analyzed in detail (Tuersuntuoheti et al., 2022).

### 2.10 MM/PBSA binding free energy calculation

The MM-PBSA approach was employed to determine the binding free energy of the THB-α-glucosidase complex. To calculate the complex’s binding free energy (DGbind), we subtracted the combined free energies of the unbound receptor (DGrec) and free ligand (DGlig) from the complex’s free energy (DGcomplex) (Lolok et al., 2022). Polar solvation energy calculations utilized the Poisson-Boltzmann equation with a 0.5 Å grid spacing. Water was chosen as the solvent, reflected by setting the dielectric constant to 80. We estimated the nonpolar component by calculating the solvent-accessible surface area, using a 1.4 Å probe radius. Our evaluation of the ligand-protein complex’s binding free energy considered four key components: van der Waals interactions (ΔG_van der Waals_), electrostatic forces (ΔG_Electrostatic_), polar solvation energy (ΔG_Polar Solvation_) and nonpolar solvation energy (ΔG_Non-Polar Solvation_).

### 2.11 ADMET (absorption, distribution, metabolism, excretion and toxicity) property prediction

To assess the ADME characteristics of THB, we utilized the Swiss ADME online platform (http://www.swissadme.ch/). The resulting predictions were then evaluated in accordance with Lipinski’s rule of five. This rule stipulates several criteria: the compound should have a molecular mass not exceeding 500 Da, a logarithm of the partition coefficient (log P) under 5, no more than five hydrogen bond donors (HB), and fewer than 10 hydrogen bond acceptors (HBA). Furthermore, we employed the admet SAR tool (http://lmmd.ecust.edu.cn/admetsar2) to forecast the toxicological profile of THB, providing a comprehensive assessment of its pharmacological potential.

### 2.12 Cytotoxicity assay

HepG2 and MDA-MB-231 cells were seeded into 96-well plates, with each well containing 8 × 10^3^ cells. The cells were allowed to adhere overnight before being subjected to THB treatment at various concentrations (0, 31.25, 62.5, 125, 250 and 500 μM) for a period of 24 h. Following the exposure, we evaluated cell viability using the cell counting kit-8 (CCK-8) assay (Meilunbio, Dalian, China). The procedure was carried out in accordance with the protocol provided by the manufacturer. To quantify the results, we measured the absorbance of each well at a wavelength of 450 nm using a microplate reader.

### 2.13 Oral sucrose tolerance experiment in mice

We obtained male ICR mice, aged 4 weeks and weighing between 20 and 25 g, from Beijing Vital River Laboratory Animal Technology Co., Ltd (Beijing, China). These SPF-grade animals were housed under controlled conditions with a 12-h light/dark cycle and had *ad libitum* access to standard pellet feed and water. A 1-week acclimatization period was provided before the start of the experiment. The study was conducted in accordance with the guidelines set by the Institutional Animal Care and Use Committee of Beijing University of Chinese Medicine.

The mice were randomly assigned to one of four groups, each consisting of six animals. Following a 12-h fasting period, the mice received oral gavage of either normal saline, acarbose (15 mg/kg), or THB at two doses (2.76 mg/kg and 8.3 mg/kg). Fifteen minutes after treatment, the animals were administered a sucrose load (3 g/kg body weight). Blood samples were collected from the tail vein at 30, 60 and 120 min post-sucrose administration. Blood glucose levels were measured using an Yuwell Performa glucometer. The overall glycemic response was quantified by calculating the area under the curve (AUC) over the 120-min period using the trapezoidal method.

### 2.14 Statistical analysis

We presented our experimental findings as mean ± standard deviation (SD). To analyze the data, we employed GraphPad Prism 8.0 software (San Diego, California). This platform allowed us to conduct one-way analysis of variance (ANOVA) and multinomial tests on our datasets. In our statistical evaluations, we considered a *p*-value less than 0.05 to be indicative of a significant difference between groups.

## 3 Results and discussion

### 3.1 Inhibition effect of THB on α-glucosidase activity

THB exhibited significant α-glucosidase inhibitory activity, as shown in Figure 1B. Notably, the inhibitory effect of THB on α-glucosidase was concentration-dependent, with increasing efficacy observed as the concentration increased from 1.57 μM to 50 μM, reaching a maximum inhibition rate of 96.50%. The IC_50_ value of 11.52 μM further underscored the potent inhibitory activity of THB against α-glucosidase. These results suggested that THB held potential as a therapeutic agent for managing diabetes and other metabolic disorders.

**FIGURE 2:**
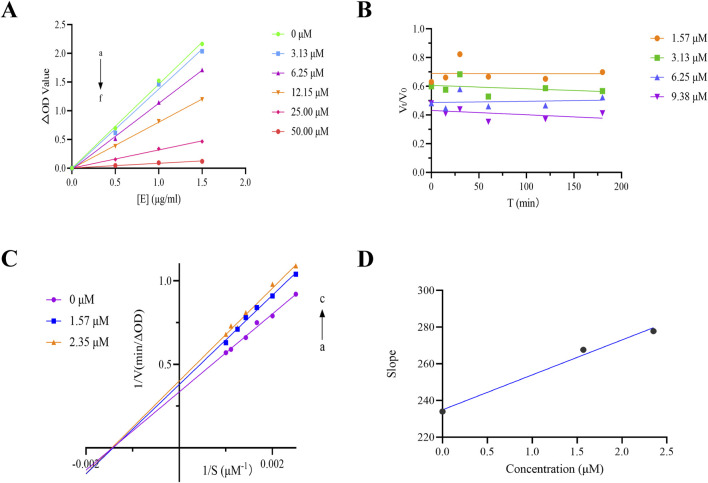
**(A)** ν vs [α-glucosidase] plots. c(pNPG) = 10 mM, c(THB) = 0, 3.13, 6.25, 12.15, 25.00, 50.00 μM for curves a → f, respectively; **(B)** Kinetic time-course of α-glucosidase inactivation measured in real-time; **(C)** Lineweaver-Burk plot for THB inhibition of α-glucosidase; **(D)** Secondary plot of THB for α-glucosidase inhibition.

To assess the reversibility of THB-induced inhibition, we plotted residual enzyme activity (v) against enzyme concentration ([E]) at different inhibitor concentrations, as shown in Figure 2A. The resulting plots exhibited linear trends that originated from the origin, with the slope decreasing as the inhibitor concentration increased. This pattern indicated that THB-induced inhibition was reversible. In contrast, irreversible inhibition would have resulted in lines corresponding to higher inhibitor concentrations maintaining the same slope as the control, intersecting the horizontal axis ([E]) at the same point.

### 3.2 Kinetics of time-course in the presence of THB on α-glucosidase inhibition

To investigate the kinetic interaction between THB and α-glucosidase, we conducted real-time monitoring of enzyme inactivation by measuring its activity at various time intervals. The results indicated that no significant changes in α-glucosidase activity were observed during incubation with THB (at concentrations ranging from 1.57 to 9.38 μM) over the specified time periods (Figure 2B). The inactivation induced by THB rapidly reached equilibrium, suggesting that THB binds to the enzyme quickly, leading to the loss of its catalytic activity without a detectable kinetic process.

### 3.3 Inhibition type of THB on α-glucosidase activity

To elucidate the inhibition mechanism of THB on α-glucosidase, Lineweaver-Burk double reciprocal plots were constructed, as illustrated in Figure 2C. The intersection point of all fitted lines was located on the x-axis. As the concentration of THB increased, the slopes of the lines progressively increased, and the Km value remained relatively constant at 0.69 ± 0.02 mM (Supplementary Table S1), while V_max_ decreased from 2.99 to 2.60 μM min^-1^. These results indicated that THB acted as a non-competitive inhibitor of α-glucosidase, suggesting its binding to the enzyme occurred at an allosteric site regardless of substrate occupancy. Unlike competitive inhibitors, which required elevated concentrations to overcome substrate competition, non-competitive inhibitors like THB are generally more specific and their effectiveness is not influenced by substrate concentration (Liu et al., 2024). Furthermore, a secondary plot of the slope versus [THB] was constructed, which exhibited a linear relationship, suggesting that THB bound to a single inhibition site on the enzyme. Additionally, the calculated Ki value of 26.26 ± 4.95 μM reflected a strong binding affinity between THB and α-glucosidase. These findings deepen our understanding of the inhibitory mechanisms of THB on α-glucosidase, offering valuable insights for future research into its biological effects.

### 3.4 Combined inhibition of THB with acarbose on α-glucosidase

**FIGURE 3:**
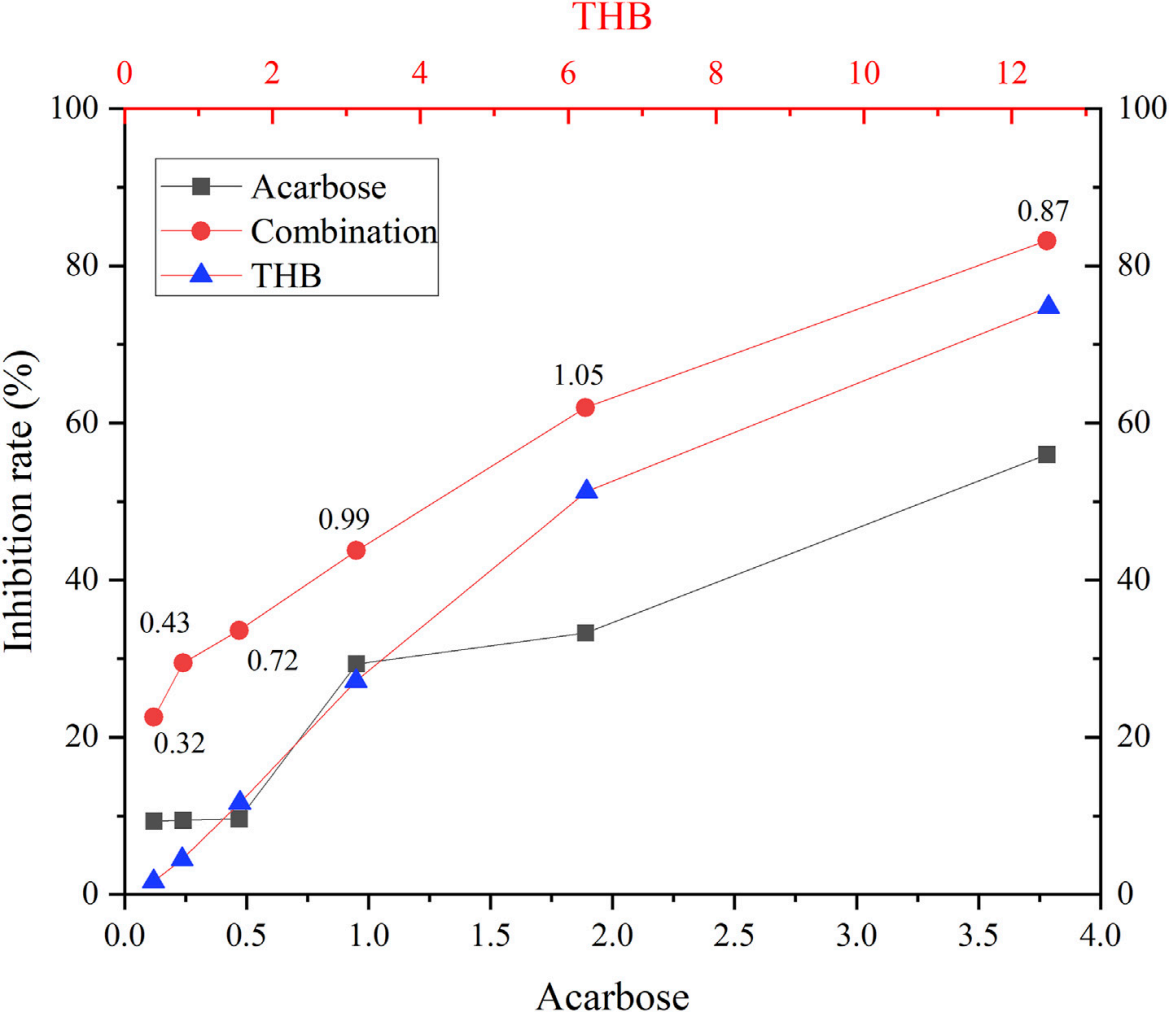
Inhibition of α-glucosidase by THB in combination with acarbose. Combination index (CI) values, above the combination line, were determined using CompuSyn software.

The synergistic inhibitory effects of THB and acarbose on α-glucosidase were evaluated using the Chou-Talalay method (Chou, 2006). As illustrated in Figure 3, all tested combinations demonstrated significantly enhanced inhibitory activity compared to individual treatments with either THB or acarbose alone. Quantitative analysis revealed CI values ranging from 0.32 to 1.05, confirming a synergistic or additive interaction between these compounds in α-glucosidase inhibition. This effect could be mechanistically explained by their distinct binding patterns on the enzyme: while acarbose, as a classical competitive inhibitor, occupied the active site of α-glucosidase, THB functioned as a non-competitive inhibitor by binding to an allosteric site. This complementary binding mechanism allowed simultaneous interaction of both inhibitors with the enzyme, resulting in enhanced suppression of its catalytic activity. Importantly, this combination not only potentiates the therapeutic efficacy of acarbose but also potentially mitigates its associated side effects through reduced dosage requirements.

### 3.5 SPR analysis

**FIGURE 4:**
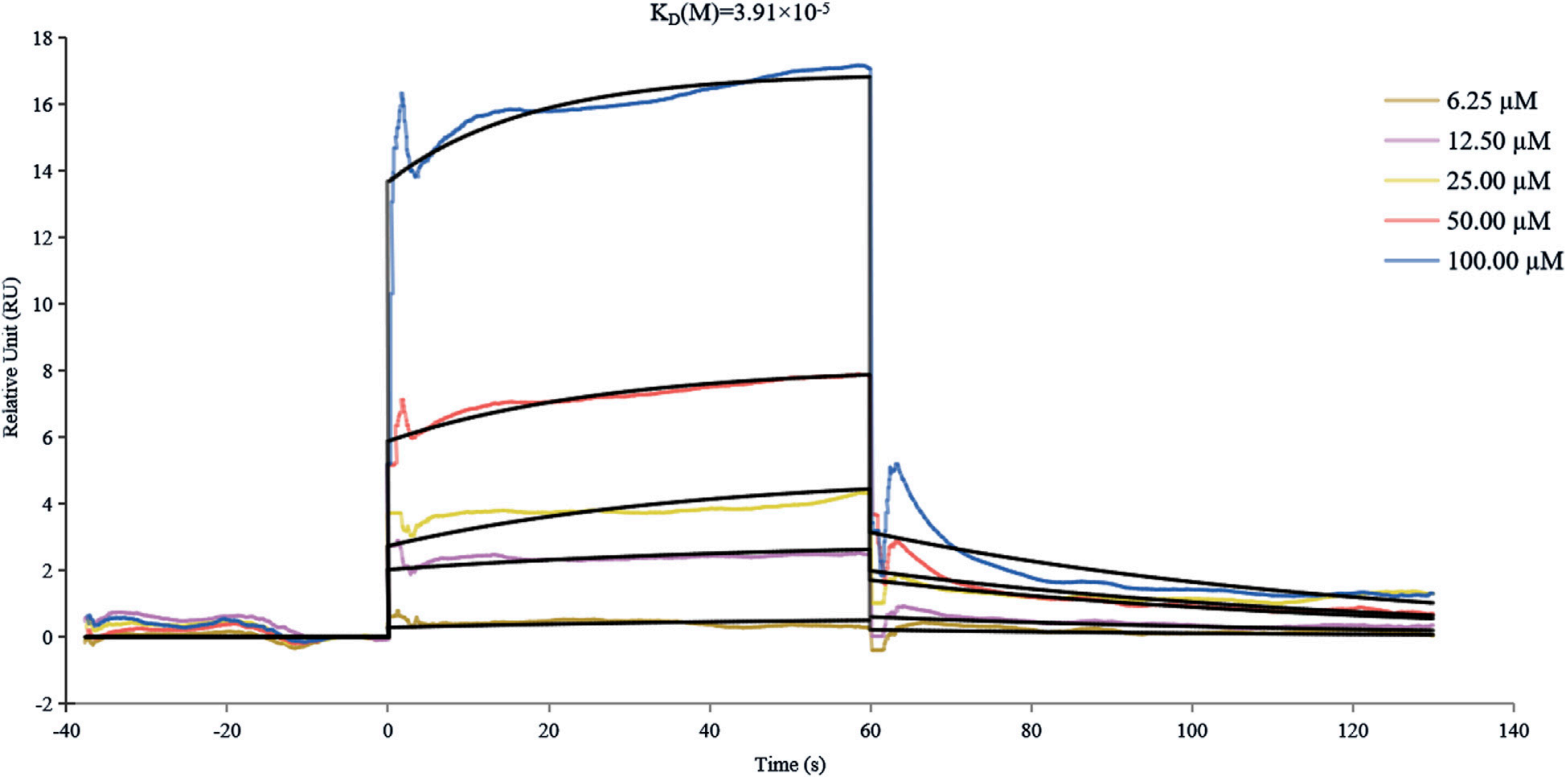
SPR analysis of the binding affinity between α-glucosidase and THB.

Surface plasmon resonance (SPR) technology has emerged as an indispensable analytical platform for biomolecular interaction studies, distinguished by its remarkable advantages, including high measurement accuracy, real-time monitoring capability, and label-free detection (Olaru et al., 2015).The SPR analysis, as illustrated in Figure 4, revealed a concentration-dependent binding profile of THB to α-glucosidase, with a K_D_ value of 3.91 × 10^−5^ M, indicating moderate binding affinity between THB and α-glucosidase. Notably, THB exhibits rapid binding to α-glucosidase, potentially facilitating immediate therapeutic effects—an essential advantage for conditions necessitating swift intervention (Zhang et al., 2024). This fast binding ensures THB promptly occupies the target site, preventing interference from competing molecules and enhancing both selectivity and efficacy. Furthermore, rapid binding may permit a reduced dosage, thereby diminishing potential side effects and minimizing toxicity risk (Xu et al., 2020).

### 3.6 Circular dichroism spectra

**FIGURE 5:**
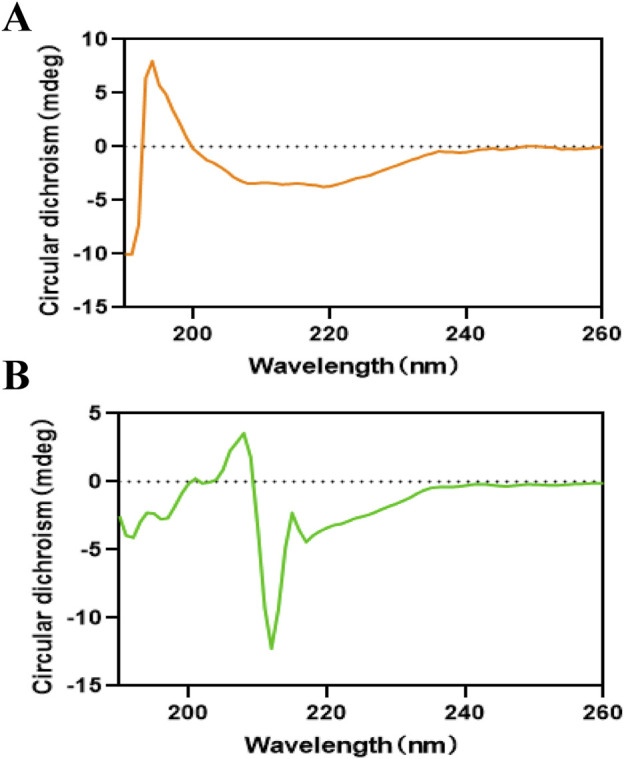
**(A)** Circular dichroism spectra of α-glucosidase; **(B)** Circular dichroism spectra of α-glucosidase with THB.

Circular dichroism (CD) spectroscopy is a powerful analytical technique for monitoring ligand-induced structural modifications in proteins. The CD spectral analysis of α-glucosidase demonstrated characteristic negative peaks at 210 nm and 216 nm (Figure 5), which were indicative of α-helical and β-sheet secondary structures, respectively. Quantitative analysis of the CD spectra using CDNN software revealed significant structural alterations upon THB binding (Supplementary Table S2). The native α-glucosidase exhibited a secondary structure composition of 14.70% α-helix, 7.80% antiparallel β-sheet, 3.00% parallel β-sheet, 27.30% β-turn, and 46.80% random coil. Following THB binding, notable structural reorganization was observed: the α-helix content decreased from 14.70% to 6.30%, while the β-turn content reduced from 27.30% to 17.30%. Concurrently, substantial increases were detected in ordered structural elements, with antiparallel β-sheet content rising from 7.80% to 19.50%, parallel β-sheet from 3.00% to 3.10%, and random coil from 46.80% to 50.10%. These structural transitions suggest a ligand-induced compaction of the enzyme’s overall conformation, potentially leading to steric hindrance at the active site. The observed structural reorganization likely impedes substrate accessibility and proper orientation within the catalytic pocket, thereby diminishing the enzyme’s catalytic efficiency. Collectively, these findings provide compelling evidence that THB binding induced significant conformational changes in α-glucosidase, ultimately modulating its enzymatic activity.

### 3.7 Molecular docking results

**FIGURE 6:**
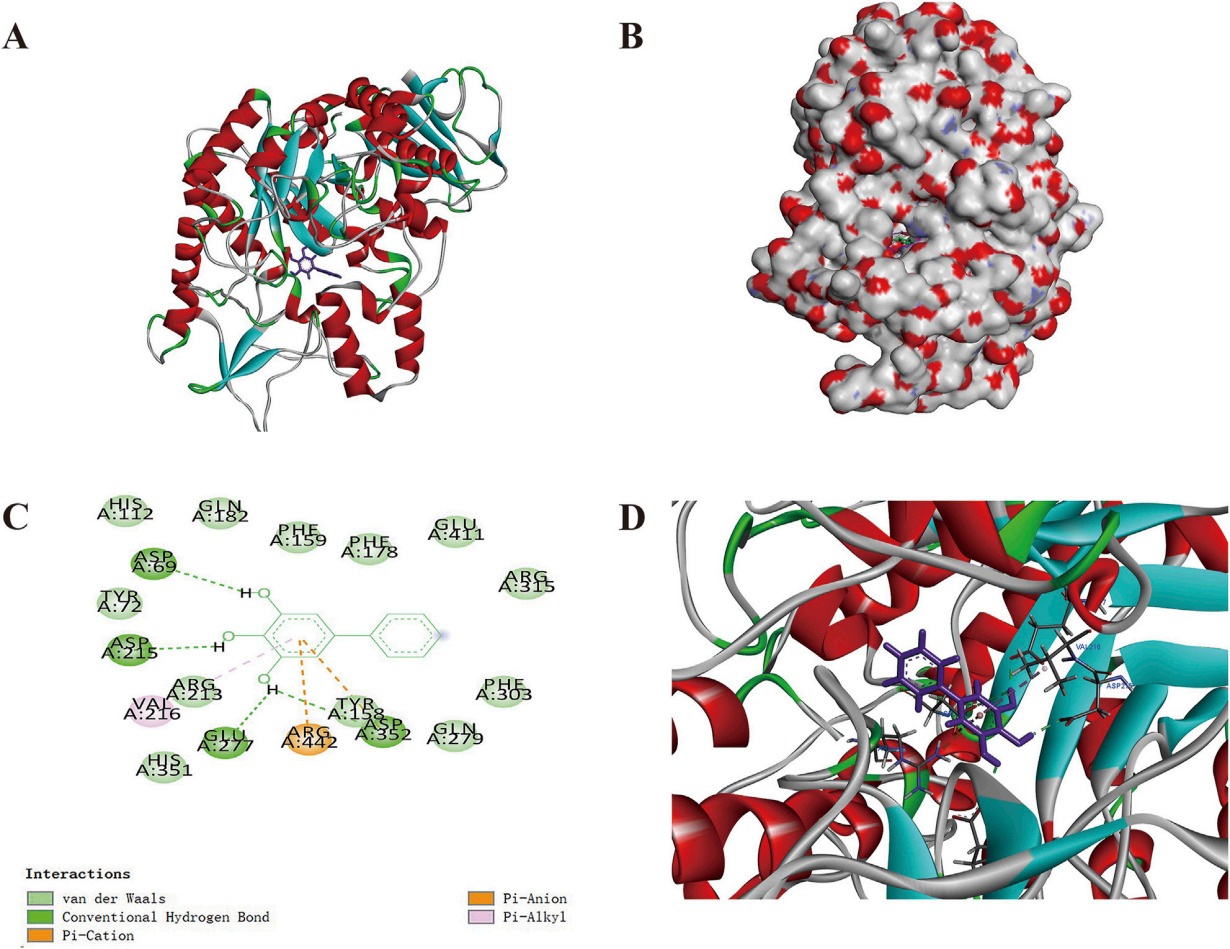
Molecular docking of THB with α-glucosidase **(A)** Structure of the THB-α-glucosidase complex; **(B)** Binding position of THB on the α-glucosidase surface; **(C)** Two-dimensional binding pattern of THB with α-glucosidase; **(D)** THB in the binding pocket.

The docking protocol was first validated through redocking of the co-crystallized ligand (maltose), yielding an RMSD of 0.9821 Å, well below the acceptable threshold of 2.0 Å (Bashyal et al., 2024). This confirmed the reliability of our computational approach for reproducing experimental binding poses. Subsequent docking of THB with *S. cerevisiae* α-glucosidase (PDB ID: 3A4A) revealed favorable binding affinities, with a docking score of −5.4340, comparable to that of the reference compound maltose (−5.9706). As shown in Figures 6A,B, THB established extensive interactions within the enzyme’s active site, including hydrogen bonds, pi-cation interactions, pi-anion interactions, pi-alkyl interactions, and van der Waals forces. Notably, four hydrogen bonds were identified between THB and the residues ASP-69, ASP-215, GLU-277 and TYR-158 (Figures 6C,D), which were likely to play a crucial role in inducing conformational changes in the enzyme.

### 3.8 MD simulations

**FIGURE 7:**
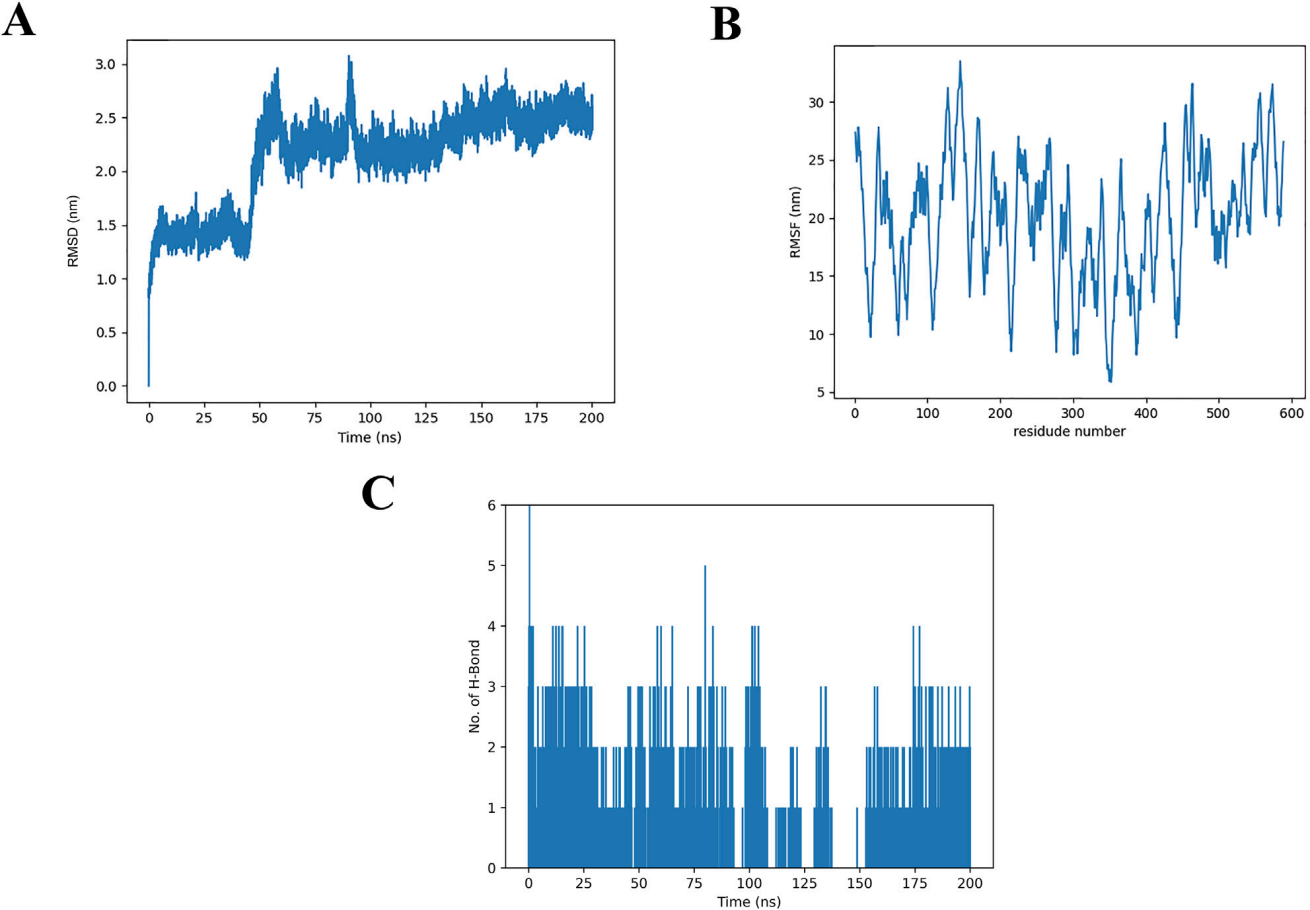
Molecular dynamics simulations of α-glucosidase and the THB complex **(A)** RMSD of α-glucosidase and its THB complex; **(B)** RMSF of α-glucosidase with THB; **(C)** Hydrogen bond formation between α-glucosidase and THB.

Considering the inherent flexibility of both the receptor and the ligand, MD simulations of the docked complexes could refine and validate the accuracy of the molecular docking results. To this end, MD simulations were performed on the optimized docking complex over a 200 ns timeframe. The stability of the protein-ligand complex was assessed by monitoring the RMSD (root mean square deviation) values of the Cα atoms relative to the protein’s initial conformation (Wong and Wong, 2024). As shown in Figure 7A, during the MD production phase (0–50 ns), the RMSD values fluctuated between 0.0 and 2.0 nm. After 50 ns, the RMSD values stabilized, displaying only minor fluctuations within the range of 2.0–3.0 nm.

As illustrated in Figure 7B, the x-axis represents the residue number, while the y-axis corresponds to the RMSF (root mean square fluctuation) values. Notably, amino acid residues within the ranges of 120–160, 450–470 and 550–580 exhibited higher RMSF values, indicating greater flexibility. These regions are likely associated with loop structures in the protein, which typically display increased mobility due to the absence of small molecule binding. In contrast, the remaining regions showed RMSF values below 30 nm, suggesting minimal conformational changes occurred during the MD simulation. This observation underscores the overall stability of the THB-protein complex, as the majority of the protein structure remained rigid and well-defined throughout the simulation.

The average number of hydrogen bonds formed between THB and α-glucosidase during the 200 ns simulation was analyzed (Figure 7C). The results revealed that THB formed up to six hydrogen bonds with α-glucosidase, with four hydrogen bonds consistently maintained throughout the 0–175 ns timeframe. These findings were consistent with the molecular docking results, highlighting the critical role of hydrogen bonding as a primary determinant in the binding of THB to α-glucosidase.

Collectively, this MD simulation study offered significant insights into the binding behavior and stability of THB with α-glucosidase. It illuminated their potential interaction mechanisms and structural characteristics, enhancing our understanding of how these molecules interacted at a molecular level.

### 3.9 Binding free energy

To further investigate the protein-ligand interactions between THB and α-glucosidase, binding free energy calculations were performed. As summarized in Supplementary Table S3, the van der Waals, electrostatic, and nonpolar solvation energies of the THB-α-glucosidase complex were all negative, indicating favorable interactions. In contrast, the polar solvation energy was positive, suggesting that polar solvation effects may partially counteract the binding of THB to α-glucosidase. Notably, the binding free energy was calculated to be −15.38 ± 1.96 kcal/mol, demonstrating that THB binds to α-glucosidase spontaneously and with high affinity. This value was significantly lower than that of honokiol (−4.9 kcal/mol), another natural biphenyl, likely due to THB’s smaller molecular size (Zhu and Zhong, 2024). These results are in excellent agreement with the findings from the earlier molecular docking and dynamics simulations, further validating the stability and affinity of the THB-α-glucosidase complex.

### 3.10 ADMET properties

The ADMET properties of compound THB were assessed using Swiss ADME and ADMET SAR tools, with detailed results provided in Supplementary Tables S4, S5. The analysis revealed that THB adhered to Lipinski’s rule of five and showed high gastrointestinal (GI) absorption, indicating favorable drug-like properties. Furthermore, THB exhibited no AMES toxicity (mutagenicity), nor any carcinogenic, hepatotoxic, nephrotoxic, respiratory, reproductive, or hemolytic toxicity, emphasizing its non-toxic profile. Cytotoxicity assays further corroborated the low toxicity of THB, showing IC_50_ values exceeding 250 μM against MDA-MB-231 and HepG2 cells after 24 h of exposure (Supplementary Figure S1). In terms of drug-drug interactions, THB was identified as a non-inhibitor of CYP2D6 and CYP3A4. This implies that THB does not interfere with the metabolism of CYP2D6 and CYP3A4 substrates. Collectively, the ADMET profile of THB highlights its potential as a promising α-glucosidase inhibitor for the treatment of diabetes, supported by its favorable pharmacokinetic and safety properties. However, these findings were derived primarily from *in silico* predictions and *in vitro* assays. To further validate THB’s safety, future studies will employ zebrafish models for rapid toxicity and target organ assessment, followed by *in vivo* evaluation in murine models.

### 3.11 Oral sucrose tolerance test

**FIGURE 8:**
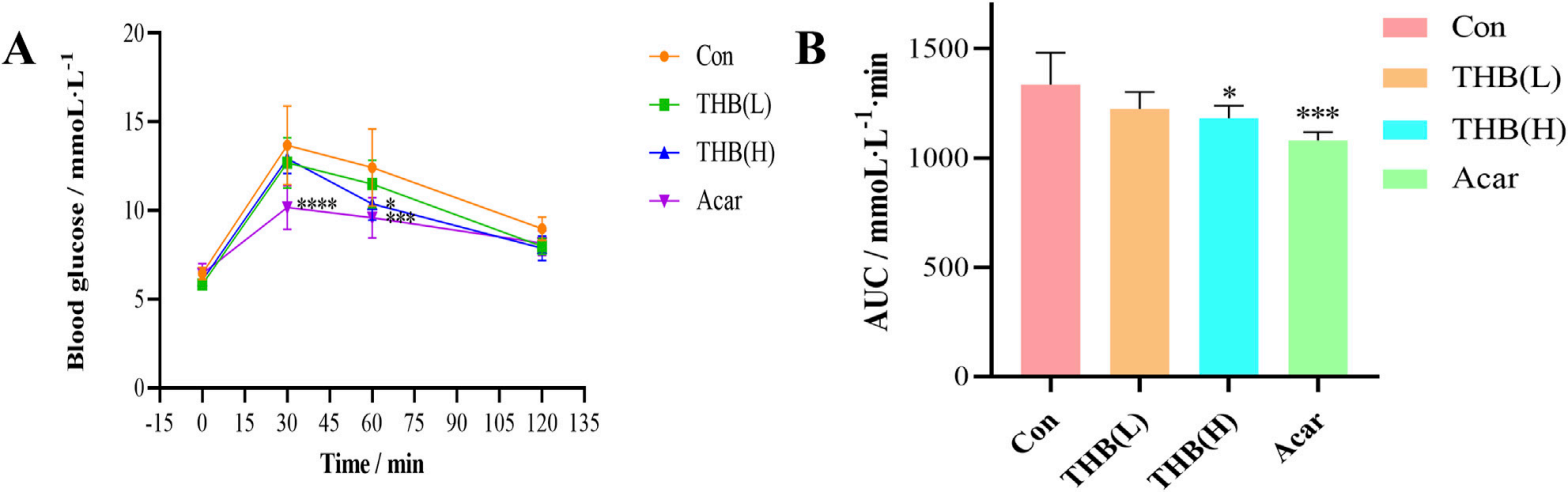
**(A)** Curves depicting PBG levels in mice following sucrose loading **(B)** Incremental area under the curve (AUC_0-120_ min) in mice after sucrose administration. Data were expressed as the mean ± SD (*n* = 6; *p < 0.05, ***p < 0.001 and ****p < 0.0001 compared to the Con group). Con, control; Acar, acarbose.

To assess *in vivo* activity, the postprandial hypoglycemic effects of THB and acarbose were evaluated using a sucrose tolerance test in mice. As illustrated in Figure 8, blood glucose levels in mice initially increased, peaked at 30 min and then gradually decreased following sucrose loading. At 60 min, the PBG levels of mice in both the high-dose THB group and the acarbose group were significantly lower than those in the control group (Figure 8A). Furthermore, the corresponding area under the curve (AUC) for glucose in mice treated with high-dose THB and acarbose was also significantly reduced compared to the control group (Figure 8B). These results demonstrated that THB effectively lowers postprandial blood glucose levels *in vivo*.

## 4 Conclusion

The study demonstrated that THB exhibited potent inhibitory activity against α-glucosidase, with an IC_50_ value of 11.52 μM. The inhibition mechanism was identified as reversible and noncompetitive. SPR analysis revealed a moderate binding affinity between THB and α-glucosidase, with a K_D_ value of 3.91 × 10^−5^ M. Notably, the combination of THB and acarbose displayed a significant synergistic inhibitory effect on α-glucosidase, attributed to their simultaneous binding to distinct regions of the enzyme. Circular dichroism analysis showed that THB binding induced specific conformational changes in the enzyme. Molecular docking and dynamics simulations further elucidated that THB interacted with the residues of α-glucosidase primarily by hydrogen bonds. This interaction induced structural modifications in α-glucosidase, leading to a substantial reduction in its catalytic efficiency. Additionally, THB was found to reduce PBG levels in mice. In conclusion, THB emerges as a promising α-glucosidase inhibitor, warranting further investigation for potential therapeutic development.

## Data Availability

The raw data supporting the conclusions of this article will be made available by the authors, without undue reservation.

## References

[B1] Bashyal J. Raut B. K. Upadhyaya S. R. Sharma K. Parajuli N. (2024). Exploration of potent human α‐glucosidase inhibitors using *in silico* approaches: molecular docking, DFT, molecular dynamics simulations, and MMPBSA. J. Chem. 16, 2086167. 10.1155/2024/2086167

[B2] Chou T. C. (2006). Theoretical basis, experimental design, and computerized simulation of synergism and antagonism in drug combination studies. Pharmacol. Rev. 58 (3), 621–681. 10.1124/pr.58.3.10 16968952

[B3] Daou M. Elnaker N. A. Ochsenkühn M. A. Amin S. A. Yousef A. F. Yousef L. F. (2022). *In vitro* α-glucosidase inhibitory activity of Tamarix nilotica shoot extracts and fractions. PLoS One 17 (3), e0264969. 10.1371/journal.pone.0264969 35286313 PMC8920278

[B4] Dirir A. M. Daou M. Yousef A. F. Yousef L. F. (2021). A review of alpha-glucosidase inhibitors from plants as potential candidates for the treatment of type-2 diabetes. Phytochem. Rev. 21 (4), 1049–1079. 10.1007/s11101-021-09773-1 34421444 PMC8364835

[B5] Du J. H. Yang D. M. Guan T. T. Wang J. Ma Y. L. Wei Z. J. (2024). Activity and mechanism of α-glucosidase inhibitory peptides released from Osmanthus fragrans seeds protein during the *in vitro* digestion. Industrial Crops Prod. 219, 119089. 10.1016/j.indcrop.2024.119089

[B6] ElSayed N. A. Aleppo G. Aroda V. R. Bannuru R. R. Brown F. M. Bruemmer D. (2023). 2. Classification and diagnosis of diabetes: standards of Care in diabetes-2023. Diabetes care 46 (Suppl. 1), S19–S40. 10.2337/dc23-S002 36507649 PMC9810477

[B7] Jiang J. Fan H. Zhou J. Qin J. Qin Z. Chen M. (2024). *In vitro* inhibitory effect of five natural sweeteners on α-glucosidase and α-amylase. Food and Funct. 15 (4), 2234–2248. 10.1039/d3fo05234f 38318730

[B8] Kaur R. Kumar R. Dogra N. Kumar A. Yadav A. K. Kumar M. (2021). Synthesis and studies of thiazolidinedione-isatin hybrids as α-glucosidase inhibitors for management of diabetes. Future Med. Chem. 13 (5), 457–485. 10.4155/fmc-2020-0022 33506699

[B9] Liu H. Wei Y. Wang Y. Zhao Q. Liu L. Ding H. (2024). Apigenin analogs as α-glucosidase inhibitors: molecular docking, biochemical, enzyme kinetic, and an *in vivo* mouse model study. Bioorg. Chem. 153, 107956. 10.1016/j.bioorg.2024.107956 39561436

[B10] Liu R. Kool J. Jian J. Wang J. Zhao X. Jiang Z. (2021). Rapid screening α-glucosidase inhibitors from natural products by at-line nanofractionation with parallel mass spectrometry and bioactivity assessment. J. Chromatogr. A 1635, 461740. 10.1016/j.chroma.2020.461740 33271429

[B11] Lolok N. Sumiwi S. A. Muhtadi A. Susilawati Y. Hendriani R. Ramadhan D. S. F. (2022). Molecular docking and molecular dynamics studies of bioactive compounds contained in noni fruit (*Morinda citrifolia* L.) against human pancreatic α-amylase. J. Biomol. Struct. Dyn. 40 (15), 7091–7098. 10.1080/07391102.2021.1894981 33682637

[B12] Luo Z. Fan H. Zhang T. Wang J. Zheng J. Guo R. (2025). A novel benzofuran derivative of β-elemene (ZT-22) inhibits hepatocellular carcinoma cell growth via directly targeting HSPA6. Chemico-Biological Interact. 415, 111514. 10.1016/j.cbi.2025.111514 40239884

[B13] Luo Z. Shi J. Jiang Q. Yu G. Li X. Yu Z. (2023). Gallic acid enhances anti-lymphoma function of anti-CD19 CAR-T cells *in vitro* and *in vivo* . Mol. Biomed. 4 (1), 8. 10.1186/s43556-023-00122-6 36871129 PMC9985527

[B14] Mushtaq A. Azam U. Mehreen S. Naseer M. M. (2023). Synthetic α-glucosidase inhibitors as promising anti-diabetic agents: recent developments and future challenges. Eur. J. Med. Chem. 249, 115119. 10.1016/j.ejmech.2023.115119 36680985

[B15] Olaru A. Bala C. Jaffrezic-Renault N. Aboul-Enein H. Y. (2015). Surface plasmon resonance (SPR) biosensors in pharmaceutical analysis. Crit. Rev. Anal. Chem. 45 (2), 97–105. 10.1080/10408347.2014.881250 25558771

[B16] Pulvirenti L. Muccilli V. Cardullo N. Spatafora C. Tringali C. (2017). Chemoenzymatic synthesis and α-glucosidase inhibitory activity of dimeric neolignans inspired by magnolol. J. Nat. Prod. 80 (5), 1648–1657. 10.1021/acs.jnatprod.7b00250 28497968

[B17] Riyaphan J. Pham D. C. Leong M. K. Weng C. F. (2021). *In silico* approaches to identify polyphenol compounds as α-glucosidase and α-amylase inhibitors against type-II diabetes. Biomolecules 11 (12), 1877. 10.3390/biom11121877 34944521 PMC8699780

[B18] Singh S. Geetha P. Ramajayam R. (2023). Isolation, synthesis and medicinal chemistry of biphenyl analogs – a review. Results Chem. 6, 101135. 10.1016/j.rechem.2023.101135

[B19] Song C. Wang X. Yang J. Kuang Y. Wang Y. Yang S. (2021). Antifungal biphenyl derivatives from Sorbus pohuashanensis leaves infected by alternaria tenuissi and their effect against crop pathogens. Chem. Biodivers. 18 (5), e2100079. 10.1002/cbdv.202100079 33821531

[B20] Tuersuntuoheti T. Pan F. Zhang M. Wang Z. Han J. Sun Z. (2022). Prediction of DPP‐IV inhibitory potentials of polyphenols existed in Qingke barley fresh noodles: *in vitro* and *in silico* analyses. J. Food Process. Preserv. 46 (10). 10.1111/jfpp.16808

[B21] Wang G. Ji Y. Li X. Wang Q. Gong H. Wang B. (2019). Utilizing the combination of binding kinetics and micro-pharmacokinetics link *in vitro* α-glucosidase inhibition to *in vivo* target occupancy. Biomolecules 9 (9), 493. 10.3390/biom9090493 31527517 PMC6770063

[B22] Wong H. Y. Wong K. B. (2024). Using AlphaFold2 and molecular dynamics simulation to model protein recognition. Methods Mol. Biol. 2841, 49–66. 10.1007/978-1-0716-4059-3_4 39115764

[B23] Xu H. Zhou J. Yu J. Wang S. Copeland L. Wang S. (2020). Revealing the mechanisms of starch amylolysis affected by tea catechins using surface plasmon resonance. Int. J. Biol. Macromol. 145, 527–534. 10.1016/j.ijbiomac.2019.12.161 31870878

[B24] Xu K. He W. Yu B. Zhong K. Zhou D. Wang D. W. (2024). Effects of different treatments for type 2 diabetes mellitus on mortality of coronavirus disease from 2019 to 2021 in China: a multi-institutional retrospective study. Mol. Biomed. 5 (1), 18. 10.1186/s43556-024-00183-1 38755442 PMC11099001

[B25] Yamamoto K. Miyake H. Kusunoki M. Osaki S. (2010). Crystal structures of isomaltase from *Saccharomyces cerevisiae* and in complex with its competitive inhibitor maltose. FEBS J. 277 (20), 4205–4214. 10.1111/j.1742-4658.2010.07810.x 20812985

[B26] Zhang R. Zhang Y. Yu T. Zhang Z. Chen Y. Jiang Z. (2024). Inhibition mechanisms of α-glucosidase by eight catechins: kinetic, molecular docking, and surface plasmon resonance analysis. Int. J. Biol. Macromol. 283 (Pt 2), 137365. 10.1016/j.ijbiomac.2024.137365 39547633

[B27] Zhu H. Zhong X. (2024). Honokiol as an α-glucosidase inhibitor. Front. Pharmacol. 15, 1425832. 10.3389/fphar.2024.1425832 38962316 PMC11220239

