# Supplementary material for: Exploring the inhibitory effect and mechanism of 3,4,5-trihydroxybiphenyl on α-glucosidase: an integrated experimental and computational approach
Source: Front Pharmacol. 2025 Jul 3;16:1584264. doi: 10.3389/fphar.2025.1584264 (PMC12287757; doi:10.3389/fphar.2025.1584264)
Supplement: Supplementary file 1 [file Supplementaryfile1.docx]

**Exploring the inhibitory effect and mechanism of 3,4,5-trihydroxybiphenyl on α-glucosidase: An integrated experimental and computational approach**

Ruofan Guo ^a, b, 1^, Guohua Yu ^c, 1^, Yuan Li ^a^, Youyou Wang ^a, f^, Huixia Fan ^f^, Shuo Zhang ^d^, Chen Wang ^e^, Junhui Zhou ^a, f^, Jian Yang ^a, f^, Feng Gao ^b, g, *^, Zhiqiang Luo ^a, *^

^a^ *State Key Laboratory for Quality Ensurance and Sustainable Use of Dao-di Herbs, National Resource Center for Chinese Materia Medica,* *China Academy of Chinese Medical Sciences, Beijing 100700, China*

^b^ *Xiyuan Hospital of China Academy of Chinese Medical Sciences, Beijing 100091, China*

^c^ *School of Life Sciences, Beijing University of Chinese Medicine, Beijing 102488, China*

*^d^ School of Traditional Chinese Medicine, Beijing University of Chinese Medicine, Beijing 102488, China*

*^e^ School of Chinese Materia Medica, Beijing University of Chinese Medicine, Beijing 102488, China*

^f^ *Evaluation and Research Center of Daodi Herbs of Jiangxi Province, Ganjiang New District 330000, China*

^g^ *Hebei Baicaokangshen Pharmaceutical Co., Ltd, Shenzhou 053800, China*

* *Corresponding Author: Feng Gao, No.1 Xiyuan Playground, Haidian District, Beijing, China. E-mail address: gf_80@163.com (F. Gao); Zhiqiang Luo, No.16, Nanxiaojie, Dongzhimennei, Dongcheng District, Beijing, China. E-mail address: luozhiqiang@nrc.ac.cn (Z. Luo).*

*^1^ These authors contributed equally to this work.*

**Table S1.** The kinetic parameters of α-glucosidase exhibit concentration-dependent variations in response to different inhibitor concentrations.

| THB (μM） | K_m_ (μM) | V_m_ (μM·min^-1^) | K_i_ (μM) |
| --- | --- | --- | --- |
| 0 | 698.91 | 2.99 | 26.26±4.95 |
| 1.57 | 706.07 | 2.82 |  |
| 2.35 | 692.72 | 2.69 |  |
| 4.69 | 664.02 | 2.60 |  |

**Table S2.** Structural characterization of ligand-free α-glucosidase and its molecular interactions with THB inhibitor through circular dichroism spectroscopy.

| Sample | Helix (%) | Antiparallel (%) | Parallel (%) | Beta-Turn (%) | Random. Coil (%) |
| --- | --- | --- | --- | --- | --- |
| α- Glucosidase | 14.70 | 7.80 | 3.00 | 27.30 | 46.80 |
| α- Glucosidase+THB | 6.30 | 19.50 | 3.10 | 17.30 | 50.10 |

**Table S3.** Energetic profiling of THB-α-glucosidase molecular interactions through molecular mechanics/Poisson-Boltzmann surface area (MM/PBSA) analysis.

| Energy components (kcal/mol) | THB |
| --- | --- |
| △G_van der Waals_ | -1.78±3.22 |
| △G_Eletrostatic_ | -41.55±4.98 |
| △G_Polar Solvation_ | 29.08±3.11 |
| △G_Non-Polar Solvation_ | -1.12±0.14 |
| △G_Binding_ | -15.38±1.96 |

| SI.No. | Properties | Lipinski Rule | THB |
| --- | --- | --- | --- |
| 1 | Molecular mass | <500 Dalton | 202.21 |
| 2 | High lipophilicity | Log P<5 | 2.48 |
| 3 | Hydrogen bond donors | <5 | 3 |
| 4 | Hydrogen bond acceptors | <10 | 3 |
| 5 | Molar refractivity | Between 40 and 130 | 57.95 |

**Table S4.** Assessment of the drug-like characteristics of compound THB based on Lipinski's rule of five.

**Table S5.** Prediction of pharmacokinetics and biosafety of compound THB.

| Models | Compound |
| --- | --- |
|  | THB |
| AMES (Mutagenicity) | No |
| GI absorption | High |
| Carcinogenicity (binary) | No |
| CYP2D6 inhibition | No |
| CYP3A4 inhibition | No |
| Hepatotoxicity | No |
| Nephrotoxicity | No |
| Respiratory toxicity | No |
| Reproductive toxicity | No |
| Hemolytic toxicity | No |


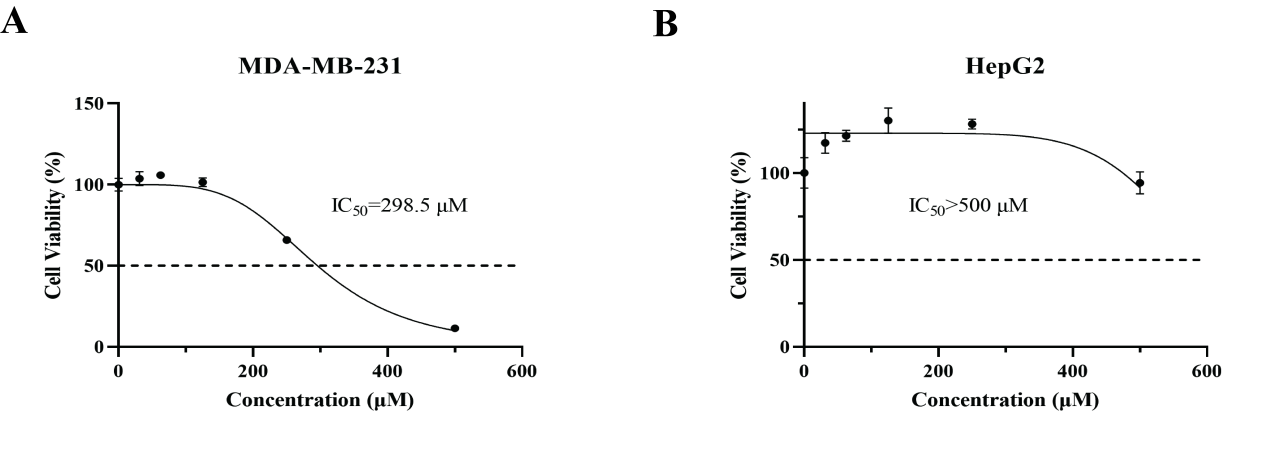


**Figure S1:** (A) MDA-MB-231 cell survival after 24h exposure to THB (0-500 μM); (B) HepG2 cell survival after 24h exposure to THB (0-500 μM)
